# Understanding the interplay between lifestyle factors and emotional distress for hemorrhagic stroke survivors and their informal caregivers: Protocol for a mixed methods dyadic natural history study

**DOI:** 10.1371/journal.pone.0261635

**Published:** 2022-01-21

**Authors:** Ethan G. Lester, Nathan S. Fishbein, Olivia Higgins, Jonathan Rosand, Ana-Maria Vranceanu

**Affiliations:** 1 Department of Psychiatry, Integrated Brain Health Clinical and Research Program, Massachusetts General Hospital, Boston, Massachusetts, United States of America; 2 Harvard Medical School, Boston, Massachusetts, United States of America; 3 Department of Neurology, Henry and Allison McCance Center for Brain Health, Massachusetts General Hospital, Boston, Massachusetts, United States of America; 4 Division of Neurocritical Care and Emergency Neurology, Department of Neurology, Massachusetts General Hospital, Boston, Massachusetts, United States of America; PLoS ONE, UNITED STATES

## Abstract

Emotional distress (depression, anxiety, and PTS) and unhealthy lifestyle factors (e.g., smoking, alcohol consumption, poor diet, limited physical activity, medication adherence) are common in hemorrhagic stroke (HS) survivors and may increase risk for recurrence, morbidity, and mortality. Emotional distress and unhealthy lifestyle factors tend to be interdependent between survivors and their informal caregivers (e.g., family and friends who provide unpaid care; together called dyads), such that one partner’s lifestyle and coping behaviors influence the other’s behaviors, yet no research has closely examined this relationship in HS dyads over time. We will conduct a mixed methods study to quantitatively and qualitatively understand the longitudinal relationship between emotional distress and lifestyle factors across time in this population (HS dyads) to identify treatment targets to prevent emotional distress chronicity and stroke recurrence. In aim 1, we will assess emotional distress (i.e., depression, anxiety, and PTS) and lifestyle factors (smoking, alcohol consumption, poor diet, limited physical activity medication adherence/blood pressure control) in dyads of survivors of HS and their caregivers (N = 80), at three separate time points (hospitalization in the Neuro-ICU, 1, and 3 months later). We hypothesize that 1) lifestyle factors and emotional distress will be interrelated within and across time for both survivors and caregivers, and 2) lifestyle factors and emotional distress will be interdependent between survivors and caregivers. We also aim to explore the nuanced interplay between lifestyle factors and emotional distress and gain in depth information on barriers and facilitators for a dyadic intervention to optimize lifestyle behaviors and emotional functioning in HS dyads. Eligible patients will be adults who have a caregiver also willing to participate. Patients will be referred for study participation by the nursing team who will ensure that they are cognitively able to meaningfully participate. Multilevel dyadic modeling (i.e., actor-partner interdependence model; APIM) with distinguishable dyads will be used to determine influences of these factors onto each other over time. In Aim 2, we will conduct live video qualitative dyadic interviews (N = 20 or until theme saturation) at all time points from the same participants with and without emotional distress and at least one lifestyle risk factor, to understand the nuanced relationships between emotional distress and lifestyle behaviors, and barriers and facilitators to engagement in a skills-based psychosocial intervention. Interviews will be analyzed using inductive and deductive approaches. The present study is currently ongoing. So far, we enrolled 2 participants. Recruitment will end October 2022 with plans to analyze data by December 2022. The findings from this study will be used to further develop psychosocial interventions and inform novel treatments for survivors of HS and their informal caregivers.

## Introduction

Hemorrhagic strokes (HS) account for less than a quarter of all strokes yet are some of the deadliest and most debilitating forms of cerebrovascular disease [1]. Despite static risk factors (e.g., age, ethnicity, education, medical/mental health history) and post-stroke medical events (e.g., delayed infarction, pneumonia, sepsis) contributing to poor overall quality of life in approximately 1/3 of survivors with HS [2], there are clear modifiable risk factors, including lifestyle (e.g., smoking, alcohol consumption, poor diet, limited physical activity, medication adherence/blood pressure control) [3–8] and emotional distress (depression [9], anxiety [10–12], and post-traumatic stress [13, 14]) which may improve health outcomes post-HS [8]. Unfortunately, HS survivors receive very little emotional-behavioral support after illness onset [2], and research is limited on the interplay between lifestyle factors and emotional distress post-stroke in this population.

Along with survivors [15–18], informal caregivers can also develop chronic psychosocial sequelae in the form of depression, anxiety and PTS [17, 19–22]. Our prior research with patients with acute brain injury hospitalized in the Neuro-ICU suggests dyadic interdependence, such that one partner’s coping and emotional function influence the other’s over time [23–25]. Research in other populations suggests a very similar dyadic interdependence for lifestyle factors [26, 27]. Although our previous studies demonstrate the benefits of a dyadic approach in an acute neurological illness population for emotional distress [23–25, 28–30], there have been no studies incorporating lifestyle and emotional distress factors simultaneously in one treatment modality, nor prospective data which specifically links these two modifiable domains to a conceptual model for treatment targets in HS dyads. Moreover, current psychosocial interventions with neurological disorders are typically focused either on survivor or caregiver outcomes and are not sensitive to the specific needs of HS dyads, which usually include greater physical and psychosocial sequelae, symptom burden, higher recurrence risk, and substantial disability, morbidity, and mortality.

Based on the limited research, a dyadic approach to improving outcomes in HS survivors and caregivers is ideal because: 1) the HS event can serve as a pivotal event whereby survivors and informal caregivers are highly motivated for immediate behavioral change; 2) informal caregivers are already involved in stroke care and can engage with interventions alongside the survivors. However, the interplay between these major modifiable factors over time, as well as the shared survivor-caregiver experiences from hospitalization through 3 months post stroke are not known.

Therefore, we aim to understand the longitudinal association between lifestyle factors and emotional distress among survivor-caregiver dyads from hospitalization in the Neuro-ICU (baseline) to 1 and 3 months later. We hypothesize that lifestyle factors and emotional distress will be interrelated within and across time for both survivors and caregivers and that these variables will be interdependent between survivors and caregivers within and across time [31]. We also aim to understand the nuanced interplay between lifestyle factors and emotional distress and gain in depth qualitative information on barriers and facilitators for a dyadic intervention to optimize lifestyle behaviors and emotional functioning in HS dyads.

## Methods

### Study design

This is a mixed methods study designed to examine the longitudinal association between lifestyle factors and emotional distress among hemorrhagic stroke survivor-caregiver dyads from hospitalization (baseline), 1 and 3 months later. The quantitative and qualitative information gathered from this research will be used to develop a dyadic psychosocial intervention to optimize recovery for patients with HS and their caregivers. All ethical guidelines have been met and ethical approval from the Massachusetts General Hospital (MGH) institutions IRB has been secured (approved 07/29/2021; Protocol # 2021P001923). Per our IRB’s informed consent policy, our research study only includes interactions involving survey procedures and thereby does not require written or oral consent if consent is implied as part of beginning the survey document. An informational fact sheet will be administered to each participant to explain the purposes of the study and outlines the level of participation requested.

### Setting

Recruitment and procedure implementation will occur at the Neuro-ICU at MGH, a northeastern academic medical facility. The Neuro-ICU at MGH sees approximately 170 survivors with HS annually (~100 ICH, ~70 SAH; based on personal correspondence with Neuro-ICU Medical Director). The MGH Neuro-ICU contains 22 patient rooms and is staffed by ~80 Neurointensive Care Nurses and a Clinical Nurse Specialist. Physician coverage is provided by two teams of fellows, residents, and board-certified Neurointensive Care attendings. Coverage is provided 24 hours per day/7 days per week by fellows and residents. Approximately 1700 patients with an acute neurological condition are seen yearly in the MGH Neuro-ICU, with approximately 170 of these admissions related to hemorrhagic stroke, ensuring participant enrollment is feasible and consistent with the study timeline.

### Inclusion/Exclusion criteria

Each member of the survivor-caregiver dyad must meet the eligibility criteria to enroll in the study. The survivor must be diagnosed with HS and admitted to the Neuro-ICU, 18 years of age or older, English speaking, have a life expectancy above 3 months, and cognitively intact as judged by the nursing team. The caregiver must be responsible for the informal aftercare of a survivor admitted, 18 years of age or older, and English speaking. Inclusion and exclusion criteria can be view in Table 1.

**Table 1:** Hemorrhagic stroke survivor and caregiver inclusion criteria for present study.

Survivor Eligibility Criteria	Caregiver Eligibility Criteria
• Diagnosis of Hemorrhagic Stroke.	• Informal caregiver to survivor admitted to Neuro-ICU for Hemorrhagic Stroke
• Admitted to Neuro-ICU.	• 18+ years of age.
• 18+ years of age.	• English speaking.
• English speaking.	
• Life expectancy above 3 months.	
• Cognitively intact as judged by the nursing team.	

### Recruitment

**Fig 1:**
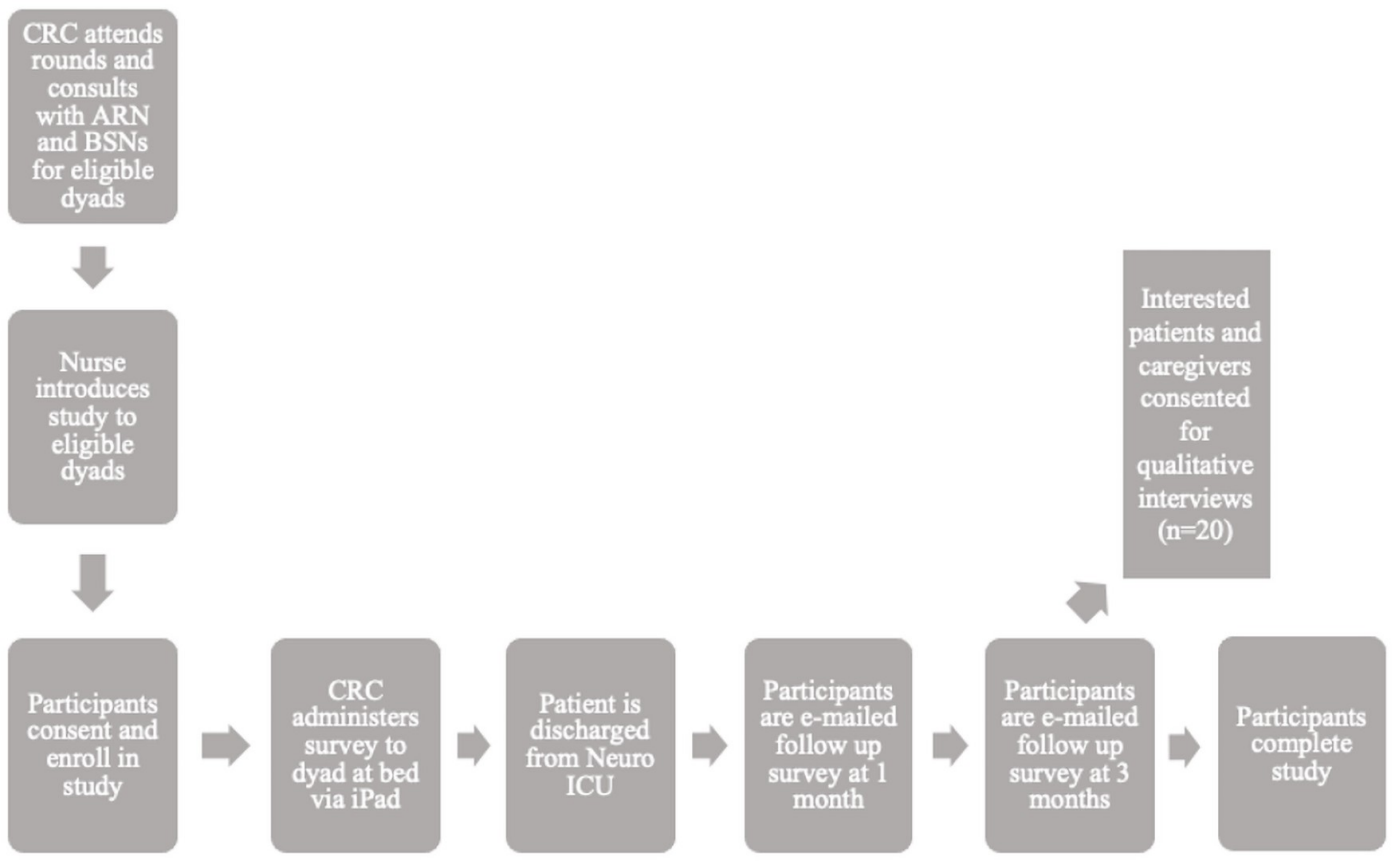
Study flow and design. CRC = Clinical research coordinator; ICU = Intensive Care Unit, ARN = Attending registered nurse, BSN = Bedside registered nurse. Above shows the flow of study recruitment. Recruitment will begin at bedside in the Neuro-ICU at MGH. We will recruit participants through referrals from the nursing team of the Neuro-ICU at MGH from morning unit rounds. Survivors who express interest in the study and their respective caregivers will meet either in person or virtually with a study research assistant to learn more about the study and be screened for eligibility. Those who wish to participate will be given information about the study as part of an informed consent process. The research assistant will administer surveys at bedside via iPad. The CRC will follow up for future surveys at 1 and 3 months. Interested caregivers will be given the opportunity to participate in qualitative interviews at 3 months.

Recruitment will occur at bedside in the Neuro-ICU at Massachusetts General Hospital (MGH). We will recruit participants through referrals from the nursing team of the Neuro-ICU at MGH from morning unit rounds. Survivors who express interest in the study and their respective caregivers will meet either in person or virtually (phone or video call for COVID-19 safety efforts if required) with a study research assistant to learn more about the study and be screened for eligibility. Those who wish to participate will be given information about the study as part of an informed consent process, as well as a resource sheet with the Principal Investigator’s (PI) contact information. These procedures (see Fig 1) will be completed in the survivor’s private hospital room. All dyads will be recruited within the first week of the survivor’s admission in the Neuro-ICU. We will recruit 80 dyads (80 survivors and 80 caregivers) over the course of 12 months. We have used this strategy successfully with previous studies within the Neuro-ICU and have had success with virtual data collection [28, 30, 32].

### Screening and enrollment

The clinical research coordinator (CRC) will obtain consent via an IRB-approved facts sheet (i.e., implied consent) delivered at time of study introduction. Participants are considered enrolled in the study when they have read over the fact sheet and begin the electronic survey if they chose to do so. The CRC will schedule a time with enrolled participants for their survey completion and potential interview in future follow-ups (one- or three-month timepoints for coordination of interview times). We will provide an additional overview of study procedures at the beginning of the interview. The study interviewer will ensure that participants understand the study procedures and that the interviewer will audio-record the interview.

### Considerations for participant safety during study participation

If a participant reports thoughts of self-harm or suicidality at any point during the study, the CRC will notify the PI. The PI will contact the participant within 24 hours to conduct a risk assessment. The PI will initiate psychiatric consults as needed. Participants who are determined to need intervention will be provided with information about resources for care. Participants who do not require higher levels of care may continue in the study. Safety will always be prioritized over study participation.

#### Assessments

For Aim 1, participants will complete online surveys through REDCap [33]. All measurements are presented below in Table 2. For the initial survey, participants may elect to either complete the surveys at bedside using an iPad or be e-mailed a secure REDCap link to complete the surveys at a later scheduled time [33]. The CRC will be available during the assessment to answer questions and provide assistance as needed/requested. For the latter two surveys, the CRC will e-mail the survey to participants via a secure REDCap links [33]. The CRC will offer to help over the phone during the latter two time points if needed/requested. If a consented participant does not complete the surveys after one week, the CRC will contact the participant via phone call and assist with completing the questionnaires over the phone. Any participants who are unreachable by the second full week (i.e., 14 days) after being e-mailed their survey link will be deemed lost to follow-up.

**Table 2:** Study measures.

Construct	Measure	Participant
Demographic	Age, gender, race, education yearly income, and marital status	Both
Functional Impact	Modified Rankin Scale [34]	Survivor only
Functional Impact	Barthel Index [34]	Survivor only
Emotional Distress	Hospital Anxiety and Depression Scale [35]	Both
Emotional Distress	Post-Traumatic Stress Checklist– 5 [36]	Both
Emotional Distress	Perceived Stress Scale [37]	Both
Emotional Distress	UCLA Loneliness Scale [38]	Both
Functional Impact	PROMIS Physical Function [39]	Both
Functional Impact	PROMIS Emotional Support [39]	Both
Functional Impact	PROMIS Social Isolation [39]	Both
Resiliency	Cognitive and Affective Mindfulness Scale [40]	Both
Resiliency	Measure of Current Status [41]	Both
Resiliency	Intimate Bond Measure [42]	Both
Resiliency	Caregiver Self-Efficacy [43]	Caregiver only
Resiliency	Preparedness for Caregiver [44]	Caregiver only
Lifestyle	Godin Leisure-Time Exercise Questionnaire [45]	Both
Lifestyle	Mediterranean Eating Questionnaire [46]	Both
Lifestyle	Pittsburgh Sleep Quality Index [47]	Both
Lifestyle	Jenkins Sleep Questionnaire [48]	Both
Lifestyle	Morisky Medication Adherence Scale [49]	Both
Lifestyle	The Fagerström Test for Nicotine Dependence [50]	Both
Lifestyle	Alcohol Use Dx Ident. Test-Consumption [9]	Both

For Aim 2, we will conduct participant interviews through secure video platform Zoom. We will audio-record, transcribe, and code the interviews. Participants will be recruited for interviews after they have completed the final survey at three months. We will attempt to recruit dyads where both participants reported at least one lifestyle risk factor and either high or low emotional distress. Given the elective nature of this interview, completion of the interview will be based on the convenience sample of those selecting to participate.

### Procedures

We will recruit 80 dyads (80 survivors and 80 caregivers) over the course of 12 months. Potential participants will be referred by the nursing team from unit rounds. The PI will give presentations to the nursing team to engage them as partners of the research and to make appropriate referrals for the study. Survivors who express interest in the study and their respective caregivers will meet either in person or virtually (phone or video call for COVID-19 safety efforts) with a CRC to learn more about the study and be screened for eligibility. Those who wish to participate will be given more information about the study as part of the consent and enrollment process. As mentioned earlier, dyads will be recruited within the first week of the survivor’s admission in the Neuro-ICU.

After determining eligibility study staff will meet with potential dyads to review the fact sheet for passive consent. After the document has been reviewed, study staff will answer any questions the participants may have. Participants will receive a description of all study procedures, information about potential risks and benefits of participation, and study contact information (including that of the IRB) in case questions arise at a later time. The fact sheet will also explicitly state that study participation is voluntary, and that participants may refuse to answer any questions that make them uncomfortable and may discontinue participation at any time. As consent is a continuous process, participants will be given a copy of the fact sheet and will be invited to ask questions about their participation at any point over the course of the study. In addition, participants will be assured that withdrawal from the study will not compromise their medical care in any way. Consent and authorization will be implied by voluntary completion of the survey (approved by the MGH IRB).

Survivors’ ability to enroll and participate will be determined based on nurses’ clinical judgments [28, 30]. For survivors unable to consent when first approached due to medical illness, we will enroll their informal caregiver and return for survivor enrollment later. Based on our previous prospective research in the Neuro-ICU, we expect the majority (~75%) of informal caregivers to be romantic/spousal partners of survivors. Only one informal caregiver will be enrolled for each survivor. If more than one informal caregiver is willing to participate, enrollment preference will be directed to the individual who is a) their spouse/partner b) the individual most responsible for their informal stroke aftercare, or c) the healthcare proxy.

Dyads will complete the initial study questionnaire in the privacy of the survivor’s hospital rooms, either using a researcher-provided iPad or their own preferred electronic device. At follow up timepoints (1 and 3 months) participants will either be emailed the link to the questionnaires or will complete the assessment over the phone with a CRC. Methods for both the baseline and follow-up data collection will be based on participant preference and ability. Dyads will have the option to complete the questionnaires in one 30-minute sittings, or over a series of meetings based on survivor related factors (e.g., fatigue, attention). The CRC for this study will assist participants with questionnaire completion as needed. We will collect clinical (e.g., relevant post-stroke complications) and demographic information from the electronic medical record throughout the study. Study procedures can be viewed in Fig 1.

### Data analysis

**Fig 2:**
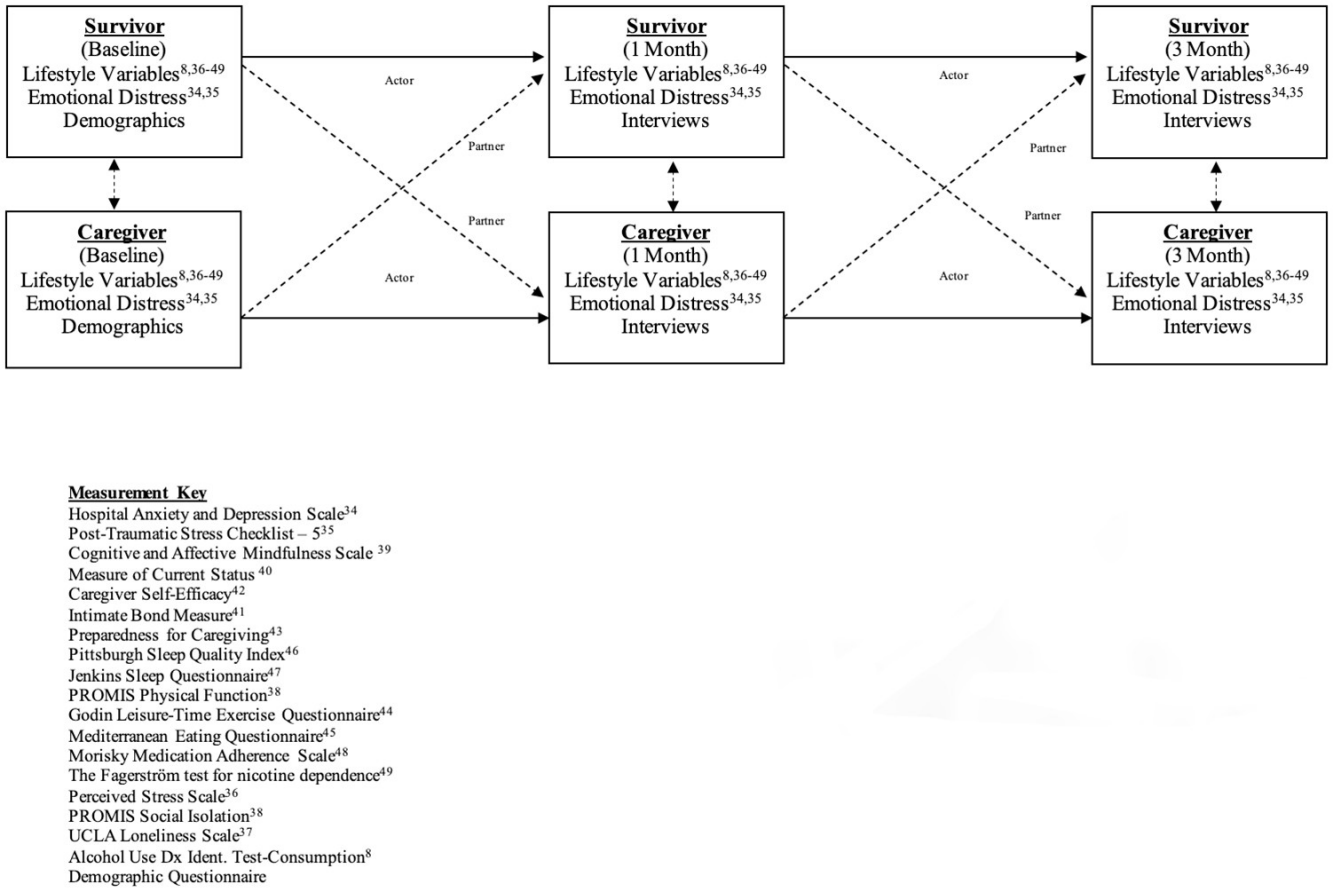
APIM design and study measures. Above describes the actor-partner interdependence model (APIM) and assessments used in our study. Multilevel dyadic modeling (i.e. APIM) with distinguishable dyads will be used to determine influences of lifestyle and emotional distress factors onto each other over time and within the dyad. We will administer lifestyle and emotional distress measures at each timepoint. In the context of this study, the actor effect is the impact of a person’s lifestyle and emotional distress factors on his/her own lifestyle and emotional distress at 1 and 3 months. Similarly, the partner effect is the impact of a person’s factors on his/her partner’s same variables at 1 and 3 months.

Preliminary analyses will be carried out to compare the relationship between psychosocial variables and demographics that will be included and/or controlled for in subsequent modeling. In aim 1, we will explore the trajectory of lifestyle factors and emotional distress from hospitalization through 3-month follow-up. Multilevel dyadic modeling (i.e., actor-partner interdependence model; APIM; See Fig 2 below) with distinguishable dyads will be used to determine influences of these factors onto each other over time and within the dyad [31, 51]. In the APIM for this study, the actor effect is the impact of a person’s lifestyle and emotional distress factors on his/her own lifestyle and emotional distress at 1 and 3 months. Similarly, their partner effect is the impact of a person’s factors on his/her partner’s same variables at 1 and 3 months. All dyad data will be restructured to a pairwise dyadic dataset. We will create grand mean centered scores that will be standardized using z-scores to obtain unstandardized and standardized regression coefficients for actor partner effects. The residual structure will be treated as heterogeneous compound symmetry in these APIM analyses [51]. In secondary analyses, we will stratify for relevant demographic (e.g., age, sex) and stroke type (e.g., subarachnoid hemorrhage [SAH], intracerebral hemorrhage [ICH]). We have successfully used these models and have published these results [23–25]. Fig 2 displays the APIM for the current study with measures and timepoints for HS dyads.

In Aim 2, we will conduct live video qualitative dyadic interviews (N = 20 or until saturation) at the same time points from participants (ICH and SAH stratified) with and without emotional distress and at least one lifestyle risk factor to understand the nuanced relationships between emotional distress and lifestyle behaviors, and barriers and facilitators to engagement in a skills-based psychosocial intervention. All qualitative data will be analyzed in NVivo 10 [52] using a hybrid inductive-deductive analytic strategy [53]. Similar to other methods we have used, we will de-identify and transcribe interviews verbatim and upload to NVivo 10. We will construct the primary codebook consistent with study goals. Transcript coders will practice coding a single transcript and we will modify the initial codebook based on usability. On future transcripts, coders will meet to reconcile any disagreements and discuss coding strategy.

Coders will start with deductive a priori themes consistent with study aims (i.e., relationship between lifestyle and emotional distress after HS) and will flexibly add new codes inductively to enhance the codebook. We will construct queries using the NVivo matrix function (i.e., crosstabs) to interpret and contextualize the qualitative data. This hybrid analytic approach will allow us to extract findings within Survivor and caregiver themes and will help us gain better understanding of the relationship between lifestyle and emotional distress after HS in survivors and informal caregivers and inform future interventions targeting these variables simultaneously.

### Data management

We will store data on encrypted and password secured computers to maximize confidentiality and security. Each subject will be assigned a unique and anonymous identifier that will be associated with all collected data including questionnaires and interviews. All paper files will be stored in a secure, locked location that is only accessible to the study team. All electronic questionnaire data will be stored on REDCap [33], a web-based, HIPAA-compliant data system available through MGH

All study staff have been trained in responsible research conduct through CITI training at MGH. The PI has trained the CRC on the importance of maintaining confidentiality and the utilization of ID numbers. Data will only be accessible only to trained study staff and will be kept under lock-and-key. Data will be identified by ID number only, and a link between names and ID numbers will be kept separately under lock and key.

## Results

The present study is currently ongoing. To date we have completed 4 surveys (*N* = 4; 2 survivors and 2 caregivers) and plan to analyze the data of 80 dyads total. We will recruit participants for interviews based on initial baseline completion data and aim to interview approximately 20 survivors and caregivers separately. We anticipate recruitment to end October 2022. We aim to have data analyses completed by December 2022 and to inform future adaptations of our dyadic resiliency trial specific to HS, emotional distress, and lifestyle factors.

## Discussion

HS survivors have high rates of unhealthy lifestyle factors (e.g., smoking, alcohol consumption, poor diet, limited physical activity, medication adherence/blood pressure control) [3–7] and emotional distress (depression, anxiety, and PTS) [10–12, 14, 54, 55], which may enhance risk for HS recurrence, morbidity, and mortality. Research in non-HS populations has demonstrated that, given the interdependency of lifestyle factors between survivors and their informal caregivers, one partner’s coping and lifestyle influence the other’s behaviors over time [26, 27]. However, to our knowledge, there has been no research on the longitudinal interplay between lifestyle behaviors and emotional distress amongst dyads after HS. But, as recovery so often requires the work of both survivors and caregivers, dyadic research is imperative to understanding those affected by HS [56]. A dyadic approach to HS is also likely to be effective in producing positive outcomes after stroke [56, 57] as lifestyle behaviors [58–60] and emotional distress [23–25, 32] are conditioned in social contexts [61]. Thus, this study protocol is designed to investigate the relationships between survivor and informal caregivers’ emotional distress and lifestyle factors over time. The findings of this study will likely play a critical role in optimizing treatments for early intervention and, ultimately, long term stroke prevention.

Our study utilizes a novel method for investigating dyadic experiences of HS and has potential to illuminate connections not only between emotional distress and lifestyle, but also amongst the long-term interdependence of these variables in dyads after stroke. This research may also inform future trials aiming to modulate emotional-behavioral variables for HS prevention. However, like all studies, there are limitations to be considered. First, our recruitment and enrollment will be representative of the patient population that our hospital broadly serves. Located in Boston, Massachusetts, MGH is an academic medical center whereby a majority of patients and families seeking care come from middle class, highly educated and white socioeconomic and racial brackets. Throughout this study, we will make explicit efforts to enroll qualified survivors who come from diverse backgrounds. Relatedly, our measures are only available in English, and therefore we are not able to assess those who do not comprehend English. Based on the patient population served, future research may choose to incorporate culturally valid measures into their assessment strategy. Last, the Neuro-ICU is a fast paced and rapidly changing environment and many participants will have competing priorities when we are attempting to recruit them for survey completion. We will be mindful of our approach and expected interruptions during our data collection. We will aim to schedule our survey times so that there will be limited interruptions for survivors and caregivers. We will make note in our participant logs of any deviations from standard administration of our survey measures and will consider these when analyzing our findings. We will also document any changes in settings (e.g., sub-acute neurological floors or rehabilitation settings) if we elect to recruit from these sites in the future.

Our protocol describes a survey-based longitudinal study aimed at investigating lifestyle factors and emotional distress in HS survivors and their informal caregivers. We will report on demographic, functional, emotional, resilience, and lifestyle factors. Our study has the unique vantage of assessing these factors directly after the HS at bedside, as well as during follow-up periods which are central to survivor and caregiver recovery. We will also gather unique qualitative data through our interviews which stand to provide novel, first-hand insights into the relationship between emotional distress and lifestyle factors after HS in caregiver-survivor relationships. This study has potential to inform future interventions which aim to address this gap in social-emotional and behavioral change in dyads as they make their recovery from HS.

## Abbreviations

APIM: Actor-partner interdependence model
CG: Caregiver
CRC: Clinical research coordinator
HS: Hemorrhagic stroke
PI: Principal investigator
RCT: Randomized controlled trial
